# Plasminogen activator inhibitor 1 may promote tumour growth through inhibition of apoptosis

**DOI:** 10.1054/bjoc.2000.1207

**Published:** 2000-04-27

**Authors:** H C Kwaan, J Wang, K Svoboda, P J Declerck

**Affiliations:** Division of Hematology/Oncology, Department of Medicine, Northwestern University Medical School, Chicago, IL, 60611, USA; Laboratory for Pharmaceutical Biology and Phytopharmacology, Faculty of Pharmaceutical Sciences, Katholieke Universiteit Leuven, E. van Evenstraat 4, Leuven, B-3000, Belgium

**Keywords:** apoptosis, plasminogen activator inhibitor 1, uPA receptor

## Abstract

Plasminogen activator inhibitor 1 (PAI-1) has been found to be a bad prognostic factor in a number of tumours but the reason has not been fully explained. The human prostate cancer cell line PC-3 and the human promyelocytic leukaemia cell line HL-60 were used in this study to determine the effect of PAI-1 on spontaneous and induced apoptosis in culture. Apoptosis was induced with camptothecin or etoposide. Addition of a stable variant of PAI-1 or wild-type PAI-1 to these cells resulted in a significant inhibition of apoptosis. In contrast, both the latent form of PAI-1 and the stable variant of PAI-1 inactivated by a specific neutralizing monoclonal antibody, or the stable variant of PAI-1 in a complex with recombinant urokinase did not inhibit apoptosis. This indicated that the inhibitory activity of PAI-1 was required for its anti-apoptotic effect but the urokinase-type plasminogen activator receptor was not involved. These findings provide an explanation for the bad prognostic correlation of high PAI-1 levels in tumours. The anti-apoptotic effect was also found in non-tumoural cells including human umbilical vein endothelial cells and the benign human breast epithelial cell line MCF-10A, suggesting that this is a novel physiologic function of PAI-1. © 2000 Cancer Research Campaign


*British Journal of Cancer* (2000) **82**(10), 1702–1708
© 2000 Cancer Research Campaign
DOI: 10.1054/ bjoc.2000.1207, available online at http://www.idealibrary.com on IDEAL®

### Plasminogen activator inhibitor 1 may promote tumour growth through inhibition of apoptosis

**HC Kwaan[1], J Wang[1], K Svoboda[1] and PJ Declerck[2]**

[1]Division of Hematology/Oncology, Department of Medicine, Northwestern University Medical School, Chicago, IL 60611, USA; [2]Laboratory for Pharmaceutical Biology and Phytopharmacology, Faculty of Pharmaceutical Sciences, Katholieke Universiteit Leuven, E. van Evenstraat 4, B-3000, Leuven, Belgium

**Summary** Plasminogen activator inhibitor 1 (PAI-1) has been found to be a bad prognostic factor in a number of tumours but the reason has not been fully explained. The human prostate cancer cell line PC-3 and the human promyelocytic leukaemia cell line HL-60 were used in this study to determine the effect of PAI-1 on spontaneous and induced apoptosis in culture. Apoptosis was induced with camptothecin or etoposide. Addition of a stable variant of PAI-1 or wild-type PAI-1 to these cells resulted in a significant inhibition of apoptosis. In contrast, both the latent form of PAI-1 and the stable variant of PAI-1 inactivated by a specific neutralizing monoclonal antibody, or the stable variant of PAI-1 in a complex with recombinant urokinase did not inhibit apoptosis. This indicated that the inhibitory activity of PAI-1 was required for its anti-apoptotic effect but the urokinase-type plasminogen activator receptor was not involved. These findings provide an explanation for the bad prognostic correlation of high PAI-1 levels in tumours. The anti-apoptotic effect was also found in non-tumoural cells including human umbilical vein endothelial cells and the benign human breast epithelial cell line MCF-10A, suggesting that this is a novel physiologic function of PAI-1. © 2000 Cancer Research Campaign

**Keywords:** apoptosis; plasminogen activator inhibitor 1; uPA receptor

The plasminogen activator system has been recognized to play an important role in tumour growth and metastasis (Dano et al, 1985; Kwaan, 1992; Andreasen et al, 1997). Urokinase-type plasminogen activator (uPA) has an adverse effect on cancer as supported by in vitro and in vivo experiments showing that a high content of uPA is mitogenic, mediates pericellular proteolysis promoting tumour cell invasion and enhances angiogenesis. PAI-1 was also found to be a bad prognostic factor in a number of tumours, including carcinoma of the breast, lung, colon, ovarian, gastric and kidney (Grondahl-Hansen et al, 1993; Janicke et al, 1993; Bashar et al, 1994; Casslen et al, 1994; Foekens et al, 1994; Kobayashi et al, 1994; Kuhn et al, 1994; Nekarda et al, 1994; Pedersen et al, 1994; Sier et al, 1994; Hofmann et al, 1996; Sugiura et al, 1999). However, when human prostate carcinoma PC-3 cells were transfected with plasminogen activator inhibitor 1 (PAI-1) gene, there was a decrease in primary tumour size and decreased metastasis in vivo (Soff et al, 1995). These conflicting results have not been fully explained. A possible explanation for this apparent controversy may be related to the source of PAI-1. Studies in which PAI-1 levels were correlated with a bad prognosis involved the evaluation of PAI-1 levels in the host, such as in surgical specimens (Foekens et al, 1994; Kuhn et al, 1994; Pedersen et al, 1994; Sier et al, 1994; Hofmann et al, 1996; Sugiura et al, 1999) or in patient's plasma (Bashar et al, 1994; Casslen et al, 1994). In contrast, when the PAI-1 level is increased in tumour cells as a result of transfection, the tumour invasiveness and metastatic potential is decreased (Cajot et al, 1990; Alizadeh et al, 1995; Soff et al, 1995). In this paper, we provide data to support the possibility that the addition of PAI-1 to tumour cells in culture, acting as increased host PAI-1, inhibits apoptosis of the tumour cells. Furthermore, this novel action of PAI-1 was also observed when PAI-1 is added to non-tumoural cells in culture.

*Received 9 September 1999*
*Revised 18 January 2000*
*Accepted 26 January 2000*

*Correspondence to:* HC Kwaan, VA Lakeside Medical Center, 333 East Huron Street, Chicago, IL 60611, USA

### MATERIALS AND METHODS

### Reagents

A stable variant of PAI-1 (PAI-1-stab), recombinant wild-type PAI-1 (PAI-1-wt) and latent PAI-1 (PAI-1-lat) were produced as described previously (Gils et al, 1996; Vleugels et al, 1998). The stable variant, containing point mutations of N150H, K154T, Q301P, Q319L and M354L, does not undergo conformational changes into the latent form (Vleugels et al, 1998). A specific monoclonal antibody with neutralizing properties towards PAI-1 activity, MA-33B8, and a monoclonal antibody against PAI-1 but not interfering with the activity, MA-19A5, were produced as described (Debrock and Declerck, 1997).

Recombinant two-chain uPA was provided by Dr Jack Henkin of Abbott Laboratories (Abbott Park, IL, USA). The complex of uPA/PAI-1 was prepared by incubating equimolar amounts of uPA and PAI-1 at 37°C for 30 min. This complex forms a lytic band of MW 110 kDa on zymography (Levenson et al, 1998).

Anti-human Fas mouse monoclonal IgM clone CH11 was purchased from Immunotech (Westbrook, ME, USA). Antibodies against domain 1 or domain 2 of uPA receptor were purchased from American Diagnostica Inc. (Greenwich, CT, USA). Camptothecin (CPT) and etoposide (VP16) and all the other chemicals were purchased from Sigma Chemical Co. (St Louis, MO, USA).

### Cell culture

The human prostate carcinoma line PC-3 was originally obtained from Dr James Kozlowski of Northwestern University (Chicago, IL, USA). The human promyelocytic leukaemia cell line HL-60 was purchased from American Type Culture Collection (Rockville, MD, USA). PC-3 or HL-60 cells were maintained in RPMI-1640 medium supplemented with 10% fetal bovine serum (FBS), 2 mmol $1^{-1}$ L-glutamine, 2.5 μg $ml^{-1}$ amphotericin B, 100 IU $ml^{-1}$ penicillin and 100 μg $ml^{-1}$ streptomycin in 5% carbon dioxide ($CO_2$) at 37°C. The benign human breast epithelial cell line MCF-10A was obtained from Dr Sigmund Weitzman of Northwestern University (Chicago, IL, USA). MCF-10A cells were cultured in Dulbecco's modified Eagles medium (DMEM)/F12 medium (Life Technologies, Inc.) supplemented with 5% horse serum, 2 mmol $1^{-1}$ L-glutamine, 2.5 μg $ml^{-1}$ amphotericin B, 100 IU $ml^{-1}$ penicillin, 100 μg $ml^{-1}$ streptomycin, 10 μg $ml^{-1}$ insulin, 100 ng $ml^{-1}$ cholera toxin, 0.5 μg $ml^{-1}$ hydrocortisone and 20 ng $ml^{-1}$ epidermal growth factor in 5% $CO_2$ at 37°C. A primary culture of human umbilical vein endothelial cells (HUVEC) was provided by Dr H William Schnaper of Northwestern University (Chicago, IL, USA). The primary HUVEC (passage 5) were maintained in RPMI-1640 medium supplemented with 10% BCS, 2 mmol $1^{-1}$ L-glutamine, 2.5 μg $ml^{-1}$ amphotericin B, 100 IU $ml^{-1}$ penicillin, 100 μg $ml^{-1}$ streptomycin, 50 μg $ml^{-1}$ gentamicin, 5 U $ml^{-1}$ sodium heparin and 0.16 mg $ml^{-1}$ endothelial cell growth supplement in 5% $CO_2$ at 37°C.

### Induction of apoptosis

### *HL-60 and PC-3 cells*

Exponentially growing HL-60 or PC-3 cells that were approximately 60% confluent were exposed to 0.15 μmol $l^{-1}$ of CPT or 50 μmol $l^{-1}$ of VP16 to induce apoptosis. HL-60 cells were treated with CPT for 2 h. PC-3 cells were treated with CPT for 24 h or with VP16 for 72 h. Then the cells were harvested and analysed for percentage of cells undergoing apoptosis.

### *MCF-10A cells*

These cells were treated with 0.5 μg $ml^{-1}$ anti-Fas antibody clone CH11 for 48 h. An irrelevant mouse IgM antibody was used as a negative control.

### *HUVEC*

These cells were kept in culture medium without serum, heparin and growth factor for 16 h.

### Identification of apoptosis with annexin V-propidium iodide staining

Annexin V and propidium iodide (PI) staining was performed according to the protocol of apoptosis detection kit from R&D Systems (Minneapolis, MN, USA). Briefly, cells were rinsed once with phosphate-buffered saline (PBS) and then resuspended in onefold binding buffer. Then, $1 \times 10^5$ cells in 100 μl were incubated with 10 μl of fluorescein-conjugated annexin V and 10 μl of PI in the dark for 10 min at room temperature. The cells then were pelleted in a microcentrifuge, resuspended in 30 μl of onefold binding buffer and smeared on slides. Cells were counted under a fluorescent microscope. Total cell counts were taken under tungsten light while the annexin V-positive (green indicating early apoptosis) and the annexin V and PI-positive cells (green and red, indicating late apoptosis) were taken under ultraviolet light. In each slide, 200–300 cells were counted. The number of stained cells was divided by the total cell number to determine the per cent apoptosis. The per cent apoptosis as described in this study includes both early and late apoptotic cells in the calculation. PAI-1 has been shown to have the same effect on early and late apoptosis. Also, we calculated the percent inhibition by PAI-1 using the following formula:

$$\frac{\text{\% apoptotic cells without PAI-1} - \text{\% apoptotic cells with PAI-1}}{\text{\% apoptotic cells without PAI-1}} \times 100$$

### TdT-mediated DNA nick end labelling with fluorescein-dUTP and flow cytometry assay

The protocol was adapted from apoptosis detection system of Promega (Madison, WI, USA). Briefly, $3 \times 10^6$ cells were washed with PBS, fixed with 1% formaldehyde for 20 min on ice and then stored in 70% ethanol at –20°C for at least 4 h. After they were incubated in equilibration buffer for 5 min at room temperature, $2 \times 10^6$ cells were labelled at 37°C in the dark for 1 h with terminal deoxynucleotidyl transferase (TdT) incubation buffer containing TdT enzyme and fluorescein-dUTP. The reaction was terminated by adding 20 mmol $l^{-1}$ EDTA. The cells were then stained for 30 min with PI solution containing 5 μg $ml^{-1}$ PI and 50 μg $ml^{-1}$ RNase. The cells were analysed with a Coulter flow cytometer. Subsequently, the green fluorescence of fluorescein-dUTP and red fluorescence of PI were measured at 520 nm and 620 nm respectively.

### DNA fragmentation analysis

Genomic DNA was extracted using easy-DNA kit from Invitrogen (San Diego, CA, USA). In brief, $10^7$ cells were fixed in 70% ethanol overnight and then resuspended in 200 μl PBS buffer. After the cells were lysed with Solution A, nucleosomal DNA was isolated with Solution B and chloroform and precipitated by adding 100% ethanol. The pellet after centrifugation was rinsed with 80% ethanol and resuspended in TE buffer. The RNA in the solution was degraded by incubation with 40 μg $ml^{-1}$ RNase at 37°C for 30 min. The amount of DNA was measured on a spectrophotometer at 260 nm, and the purity of DNA was determined by an absorbance ratio of greater than 1.8 between 260 nm and 280 nm. Equal amounts of DNA (40 μg) and 10 μl low molecular weight DNA marker (Bio-Rad, Hercules, CA, USA) were loaded on a 2% agarose gel and electrophoresis for 2 h. The gel was stained with ethidium bromide. The DNA laddering was visualized by UV transillumination.

### PAI-1 activity assay

PAI-1 activity assay was assayed with a Spectrolyse™ tPA/PAI activity assay kit from American Diagnostica Inc. (Greenwich, CT, USA). Briefly, the conditioned media was incubated with tPA standard for 15 min to allow the tPA-PAI-1 reaction. Then acetate buffer was added and incubated at 37°C for 15 min to destroy $\alpha_2$-antiplasmin. The remaining tPA activity in the solution was assayed in a 96-well plate. In each well, 10 μl solution was mixed with 250 μl tissue activator reagent containing 0.04 mg $ml^{-1}$ plasminogen and 0.4 mmol $l^{-1}$ chromogenic plasmin substrate

*© 2000 Cancer Research Campaign*

Spectrolyse™ PL in Tris buffer. Then, 10 µl fibrin stimulator Desafib™ were added to accelerate the reaction. After incubation at 37°C for 75 min, the reaction was stopped by mixing with 25 µl stop solution. Absorbance at 405 nm was assayed with a plate reader. The PAI-1 activity in the conditioned media was calculated according to the remaining tPA activity in the solution determined with tPA standard curve.

### Statistical analysis

All the results represent mean value ± standard deviation. Differences in mean values were analysed using Student's *t*-test. *P*-values > 0.05 were considered to be of no statistical significance.

### RESULTS

### Effect of PAI-1 on spontaneous and induced apoptosis

HL-60 cells were cultured for 4 h to determine the per cent of spontaneous apoptosis, 12 ± 3.2. When 20 ng $ml^{-1}$ of PAI-1-stab was added, the per cent of apoptosis dropped to 7.3 ± 1.8 ($P < 0.005$). When HL-60 cells were cultured with 0.15 µmol $l^{-1}$ CPT for 4 h, the induced rate of apoptosis was 53 ± 7.9. PAI-1-stab was also able to significantly ($P < 0.005$) inhibit the CPT-induced apoptosis to 24 ± 5.8 when it was added concurrently with CPT. To insure that the PAI-1-stab was acting on the cells and not directly on the CPT to inhibit apoptosis, HL-60 was first treated with and without CPT for 2 h and then washed. Then 20 ng $ml^{-1}$ of PAI-1-stab were added and the cells were further incubated for 2 h. Figure 1 shows that PAI-1-stab is able to significantly reduce both spontaneous and CPT-induced apoptosis in these cells ($P < 0.001$). Also, PAI-1-stab is able to inhibit apoptosis in a dose-responsive manner as illustrated in Figure 2. As the concentration of PAI-1-stab was increased, the per cent inhibition increased.

PAI-1-stab can also inhibit apoptosis in PC-3 cells. As seen in Figure 3A, PAI-1-stab significantly reduces the spontaneous and CPT-induced apoptosis for these cells after 24 h of treatment ($P < 0.001$). When these cells are treated with etoposide (VP16) for 72 h to induce apoptosis, a similar effect of PAI-1-stab was found as shown in Figure 3B ($P < 0.05$).

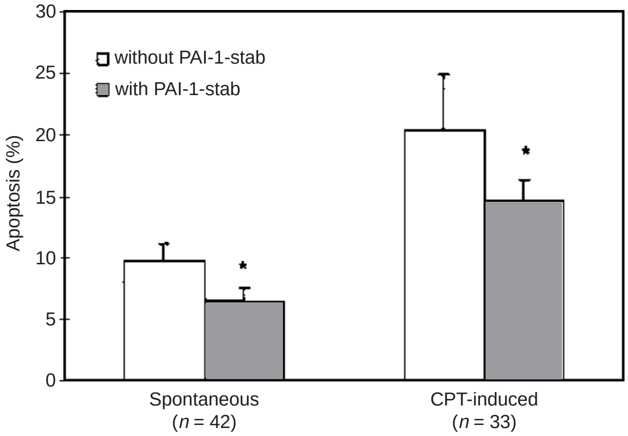

**Figure 1** PAI-1-stab inhibited spontaneous and CPT-induced apoptosis in HL-60 cells. HL-60 cells were treated with or without 0.15 µmol $l^{-1}$ CPT for 2 h, washed and then treated with 20 ng $ml^{-1}$ of PAI-1-stab for 2 h. There was a significant difference between cells with and without PAI-1 treatment for spontaneous and induced apoptosis respectively (*$P < 0.001$)

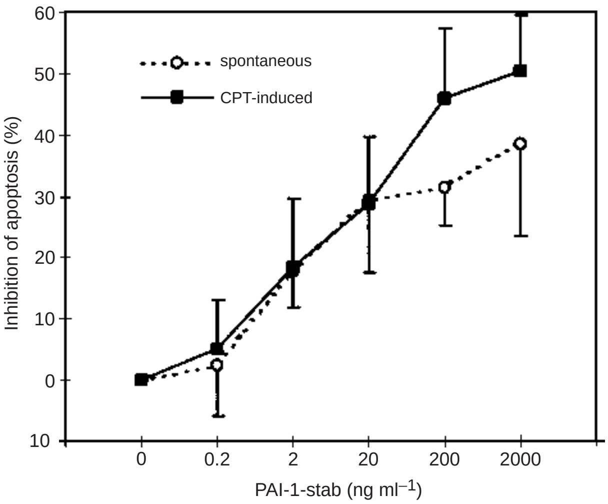

**Figure 2** PAI-1-stab inhibited apoptosis in a dose-responsive manner in HL-60 cells. The percent inhibition of spontaneous apoptosis or CPT-induced apoptosis was determined as shown in Materials and Methods, $n = 3$

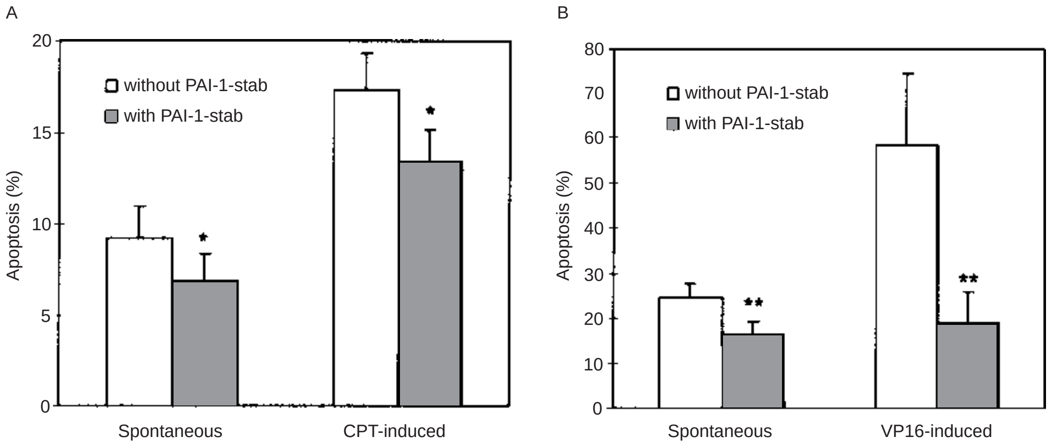

**Figure 3** PAI-1-stab inhibited spontaneous and induced apoptosis in PC-3 cells. (**A**) PC-3 cells were treated with 0.15 µmol $l^{-1}$ CPT and 20 ng $ml^{-1}$ PAI-1-stab concurrently for 24 h. There was a significant difference between the cells with and without PAI-1 treatment for spontaneous and CPT-induced apoptosis respectively (*$P < 0.001$, $n = 7$). (**B**) PC-3 cells were treated with 50 µmol $l^{-1}$ VP16 and 20 ng $ml^{-1}$ PAI-1-stab concurrently for 72 h. There was a significant difference between the cells with and without PAI-1 treatment for spontaneous and VP16-induced apoptosis respectively (**$P < 0.05$, $n = 3$)

*© 2000 Cancer Research Campaign*

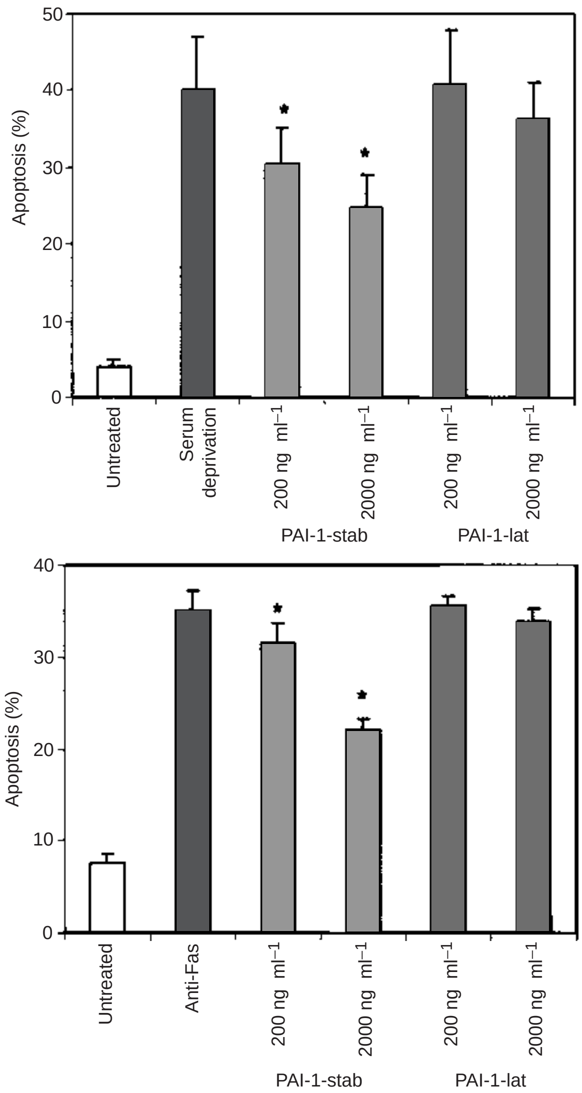

**Figure 4** (**A**) Serum deprivation-induced apoptosis in HUVEC, was inhibited by PAI-1-stab in concentrations of 200 ng ml$^{-1}$ and 2000 ng ml$^{-1}$ (*$P$ < 0.05, $n$ = 3), while latent PAI-1 in the same concentrations has no effect ($P$ > 0.05, $n$ = 3). (**B**) Apoptosis was induced in the benign breast epithelial cell (MCF-10A) by 0.5 mg ml$^{-1}$ anti-Fas antibody for 48 h. PAI-1-stab was also able to inhibit the apoptosis (*$P$ < 0.05, $n$ = 3), while latent PAI-1 has no effect ($P$ > 0.05, $n$ = 3)

Likewise, PAI-1-stab was found to inhibit apoptosis in HUVEC and MCF-10A ($P$ < 0.05), while the latent form of PAI-1 did not ($P$ > 0.05, Figure 4).

The apoptosis-inhibiting effect of PAI-1-stab was verified by DNA fragmentation analysis (Figure 5). We also found that 20 ng ml$^{-1}$ of PAI-1-stab could partially inhibit DNA laddering in apoptotic cells, while PAI-1-stab in 2000 ng ml$^{-1}$ had a greater effect. Supporting data were obtained using the TdT (TUNEL) method to stain HL-60 cells and analyse them with flow cytometry (Figure 6).

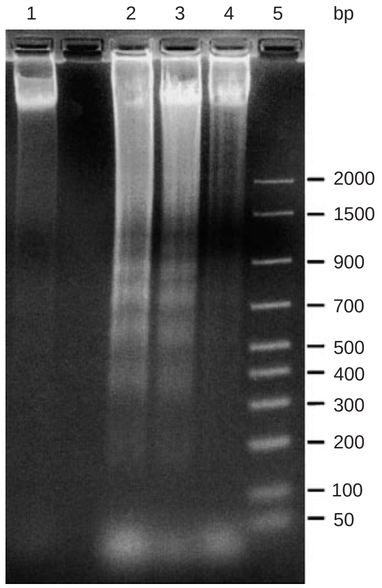

**Figure 5** PAI-1-stab inhibited CPT-induced DNA fragmentation in HL-60 cells. HL-60 cells were treated with 0.15 µmol l$^{-1}$ CPT with or without PAI-1-stab for 4 h. Lane 1: untreated, lane 2: CPT, lane 3: CPT + PAI-1-stab (20 ng ml$^{-1}$), lane 4: CPT + PAI-1-stab (2000 ng ml$^{-1}$), lane 5: molecular weight marker

### The domain of PAI-1 responsible for inhibition of apoptosis

PAI-1-wt was also tested for its effect on apoptosis. PAI-1-wt could significantly inhibit spontaneous ($P$ < 0.001) and CPT-induced ($P$ < 0.002) apoptosis in HL-60 cells. However, PAI-1-lat did not show any significant inhibition. The inhibition by PAI-1-wt was less than that of PAI-1-stab (Figure 7). To investigate this reduced effect of PAI-1-wt, a chromogenic functional activity assay was performed. The ability of PAI-1-stab to inhibit tPA activity is approximately twofold that of PAI-1-wt in the same concentration. After a 2-h incubation with HL-60 cells, the activity of PAI-1-wt decreased while the activity of PAI-1-stab remained the same (data not shown). It has been reported that the half-life of the PAI-1-wt is approximately 2 h while the half life for PAI-1-stab is 145 h (Vleugels et al, 1998). These data suggests that only the active form of PAI-1 has an apoptosis-inhibiting function.

When a specific monoclonal antibody to PAI-1, MA-33B8, was added to HL-60 cells concurrently with PAI-1-stab, the apoptosis-inhibiting function of PAI-1-stab was effectively blocked. This PAI-1 neutralizing monoclonal antibody, MA-33B8, acted in a dose-responsive manner, but a non-neutralizing antibody MA-19A5 had no such action (Figure 8). It has been suggested that MA-33B8 inactivates PAI-1-stab through acceleration of the conversion of the active conformation to the latent conformation (Vleugels et al, 1998; Verhamme et al, 1999). Thus the reactive site loop in PAI-1 may be responsible for the apoptosis-inhibiting function.

### The role of uPA receptor in the inhibition of apoptosis by PAI-1

To investigate the pathway by which PAI-1 inhibits apoptosis, the possible role of PAI-1 interaction with the uPA receptor (uPAR) was studied. One possible pathway is that PAI-1 inhibits apoptosis by binding to uPA and forming a complex. This complex then binds to domain 1 of uPAR. Figure 9A shows that the uPA/PAI-1-stab

*© 2000 Cancer Research Campaign* 

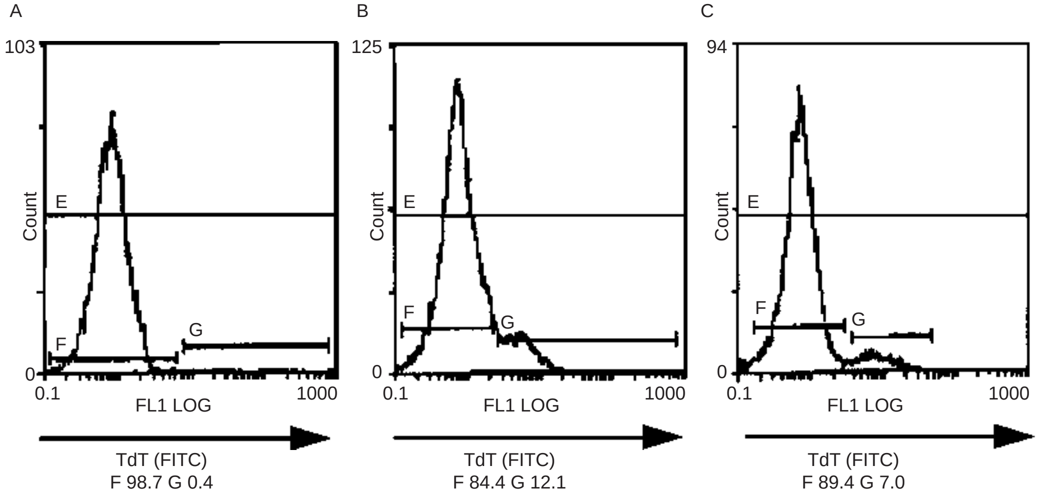

**Figure 6** Effect of PAI-1-stab on TdT staining in HL-60 cells. 20 ng ml$^{-1}$ of PAI-1-stab reduced the amount of DNA fragmentation as represented by TdT staining. Using flow cytometry, FITC staining was assayed in (**A**) untreated, (**B**) CPT-treated and (**C**) CPT + PAI-1-stab-treated cells

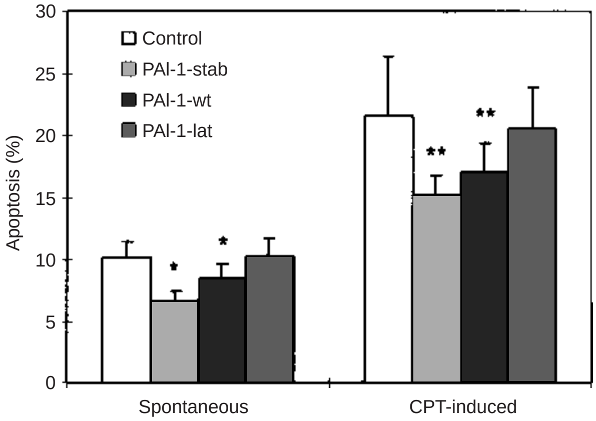

**Figure 7** The effect of 20 ng ml$^{-1}$ of PAI-1-stab, PAI-1-wt or PAI-1-lat on spontaneous and CPT-induced apoptosis in HL-60 cells. Control experiments were performed without treatment of PAI-1 in any form. PAI-1-stab and PAI-1-wt inhibited apoptosis, while PAI-1-lat had no such effect. There was a significant difference between the cells with and without PAI-1-stab or PAI-1-wt treatment for spontaneous apoptosis (*$P < 0.001$, $n = 7$) and for CPT-induced apoptosis (**$P < 0.002$, $n = 7$) respectively complex has no effect on spontaneous or on CPT-induced apoptosis in HL-60 cells. This suggests, first, that PAI-1 is not inhibiting apoptosis through binding uPA and then signalling through domain 1 of the uPAR. This is confirmed by the findings that the monoclonal antibody to domain 1 also had no effect on the apoptosis-inhibiting action of PAI-1 (Figure 9B).

Since vitronectin can bind to domain 2 of the uPAR, a monoclonal antibody to domain 2 was used to block this signal transduction pathway. The antibody did not block the function of PAI-1-stab, which suggested that PAI-1-stab did not act through binding vitronectin and then signalling through domain 2 of uPAR to inhibit apoptosis (Figure 9B).

### DISCUSSION

Tumour growth involves both cell proliferation and cell death, the latter achieved mainly through the process of apoptosis. Although the relationship between apoptosis and the plasminogen activator system is not known, our present findings showed that PAI-1 could inhibit both spontaneous and induced apoptosis in tumour cells.

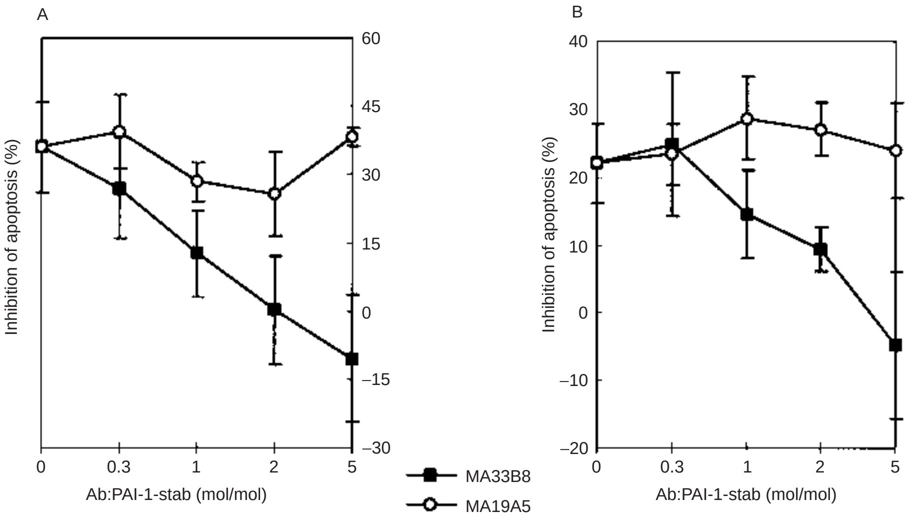

**Figure 8** The effect of monoclonal antibodies on the ability of PAI-1-stab to inhibit (**A**) spontaneous and (**B**) CPT-induced apoptosis in HL-60 cells. A PAI-1 neutralizing antibody MA-33B8 or a non-neutralizing antibody MA-19A5 was added concurrently with 20 ng ml$^{-1}$ PAI-1-stab for 2 h, $n = 3$

© 2000 Cancer Research Campaign

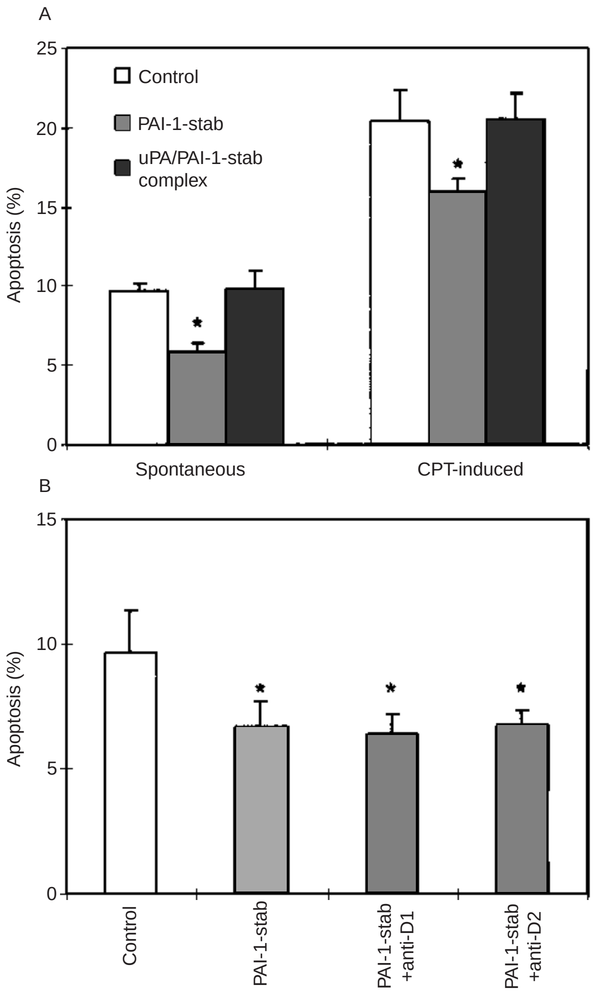

**Figure 9** (**A**) The lack of effect of uPA/PAI-1-stab complex on spontaneous and CPT-induced apoptosis in HL-60 cells. uPA and PAI-1-stab were allowed to form a complex at 37°C for 30 min. Control experiments (open bars) were performed without treatment of PAI-1-stab or uPA/PAI-1-stab complex. There was a significant difference between cells with and without PAI-1-stab treatment (shaded bars) for spontaneous and CPT-induced apoptosis. (*$P < 0.01$, $n = 4$). However, 20 ng ml$^{-1}$ of uPA/PAI-1-stab complex (solid bars) showed no inhibition of apoptosis. (**B**) The lack of effect of antibodies to domain 1 (anti-D1) or domain 2 (anti-D2) of uPAR on the apoptosis-inhibiting function of PAI-1-stab. After being incubated with 18 μg ml$^{-1}$ antibodies for 1 h, the cells were treated with 20 ng ml$^{-1}$ PAI-1-stab for 2 h (*$P < 0.005$, $n = 5$)

It has been found that PAI-1 plays an important role in migration and metastasis of tumour cells. PAI-1 may promote tumour cells invasion by regulating uPAR-dependent cell adhesion (Deng et al, 1996; Waltz et al, 1997). On the other hand, PAI-1 may inhibit integrin- and vitronectin-mediated cell migration by blocking $\alpha_v\beta_3$ binding to vitronectin (Stefansson and Lawrence, 1996; Kjoller et al, 1997). Recently Bajou et al (1998) demonstrated that deficient PAI-1 expression in host mice prevented cancer invasion and vascularization.

PAI-1, the principal inhibitor of tPA and uPA, is produced by hepatocytes, endothelial cells and many tumour cells. When first secreted by cells, it is in an active form, but readily undergoes a conformational change into the non-reactive latent form (Sancho et al, 1995; Egelund et al, 1997). In the present study, a stable variant of recombinant PAI-1 was able to significantly inhibit apoptosis in HL-60 and PC-3 cells. In addition, when PAI-1-wt was added to HL-60 cells, it could inhibit apoptosis as well. In contrast, the latent form was found to have no inhibitory effect on apoptosis.

A specific neutralizing monoclonal antibody was previously shown to accelerate conformational changes of PAI-1 resulting in loss of plasminogen activator inhibitory function of PAI-1 (Debrock and Declerck, 1997). The apoptosis-inhibiting function of PAI-1 can also be effectively blocked with this antibody. This provided additional evidence that only the active form of PAI-1 could inhibit apoptosis. Further support comes from our finding that the uPA/PAI-1 complex could not inhibit apoptosis. These data suggest that the reactive site loop in the PAI-1 molecule may be responsible for the apoptosis-inhibiting function.

The pathway by which PAI-1 signals the cell to inhibit apoptosis is not clear. One possibility is through uPAR, which is well expressed in both HL-60 and PC-3 cells. uPA is a ligand to domain 1 of this receptor and was found in the present study not to inhibit apoptosis. In fact, with amounts of uPA of 500 ng per $10^6$ cells, apoptosis was found to be enhanced to a small degree (data not shown). Domain 1 of uPAR was ruled out as a possible pathway for the action of PAI-1 because the uPA/PAI-1 complex could not inhibit apoptosis, and antibody to domain 1 did not block the action of PAI-1. Since vitronectin can also act as a ligand of uPAR by binding to domain 2 (Wei et al, 1994; Kanse et al, 1996), a monoclonal antibody to domain 2 was used to investigate the function of domain 2 in uPAR. It had no effect on the apoptosis-inhibitory effect of PAI-1. This suggests that the apoptosis-inhibitory function of PAI-1 exerts through other pathways.

This novel function of PAI-1 is not limited to tumour cells in vitro. The present findings of the inhibition of apoptosis in HUVEC and in MCF-10A cells by PAI-1 suggest that this function is also a physiologic one. Furthermore, apoptosis was induced in these benign cells by physiologic means, and not by chemotherapeutic agents.

By inhibiting apoptosis in tumour cells, PAI-1 can potentially increase the aggressiveness of the tumour, supporting the previous observations that PAI-1 is a poor prognostic indicator (Foekens et al, 1994; Kuhn et al, 1994; Pedersen et al, 1994; Sier et al, 1994; Hofmann et al, 1996; Sugiura et al, 1999). On the other hand, when PAI-1 expression is increased within tumour cells as a result of transfection, the tumour invasiveness and metastatic potential is decreased (Cajot et al, 1990; Alizadeh et al, 1995; Soff et al, 1995). These two sets of findings are not necessarily in conflict. While the transfection experiments deal with a high expression of PAI-1 by the tumour cells, the present results are those of the exogenous addition of PAI-1 to cells. It shows that PAI-1 when expressed inside the tumour cells may have different results from those when PAI-1 is high outside the cells, either added experimentally or when provided by stromal or supporting cells, such as the tumour vasculature.

In conclusion, our study demonstrates that the presence of exogenous PAI-1 can inhibit the apoptotic process. This provides an explanation why PAI-1 levels of the tumour-bearing host are associated with a bad prognosis. The decreased cancer invasion observed in experimental models using PAI-1 deficient transgenic mice (Bajou et al, 1998) may, at least in part, be explained by an increased apoptosis consequent to the absence of PAI-1. Together with these previous observations, our data also indicates that suppression of PAI-1 activity may have beneficial effects in cancer treatment.

*© 2000 Cancer Research Campaign*

### REFERENCES

Alizadeh H, Ma D, Berman M, Bellingham D, Comerford SA, Gething MH, Sambrook JF and Niederkorn JY (1995) Tissue-type plasminogen activator-induced invasion and metastasis of murine melanomas. *Curr Eye Res* **14**: 449–458

Andreasen PA, Kjoller L, Christensen L and Duffy MJ (1997) The urokinase-type plasminogen activator system in cancer metastasis: a review. *Int J Cancer* **72**: 1–22

Bajou K, Noel A, Gerard RD, Masson V, Brunner N, Holst-Hansen C, Skobe M, Fusening NE, Carmeliet P, Collen D and Foidart JM (1998) Absence of host plasminogen activator inhibitor 1 prevents cancer invasion and vascularization. *Nat Med* **4**: 923–928

Bashar H, Urano T, Pietrazek MH, Hata M, Suzuki K, Kawabe K, Takada Y and Takada A (1994) Plasminogen activators and plasminogen activator inhibitor 1 in urinary tract cancer. *Urol Int* **52**: 4–8

Cajot JF, Bamat J, Bergonzelli GE, Kruithof EKO, Medcalf RL and Testuz J (1990) Plasminogen activator inhibitor type 1 is a potent natural inhibitor of extracellular matrix degradation by fibrosarcoma and colon carcinoma cells. *Proc Natl Acad Sci USA* **87**: 6939–6943

Casslen B, Bossmar T, Lecander I and Astedt B (1994) Plasminogen activators and plasminogen activator inhibitors in blood and tumor fluids of patients with ovarian cancer. *Eur J Cancer* **30**: 1302–1309

Dano K, Andreasen PA, Grondahl-Hansen J, Kristensen P, Nielsen LS and Skriver L (1985) Plasminogen activators, tissue degradation, and cancer. *Adv Cancer Res* **44**: 139–266

Debrock S and Declerck PJ (1997) Neutralization of plasminogen activator inhibitor-1 inhibitory properties: identification of two different mechanisms. *Biochim Biophys Acta* **1337**: 257–266

Deng G, Curriden SA, Wang S, Rosenberg S and Loskutoff DJ (1996) Is plasminogen activator inhibitor-1 the molecular switch that governs urokinase receptor-mediated cell adhesion and release? *J Cell Biol* **134**: 1563–1571

Egelund R, Schousboe SL, Sottrup-Jensen L, Rodenburg KW and Andreasen PA (1997) Type-1 plasminogen-activator inhibitor: conformational differences between latent, active, reactive-centre-cleaved and plasminogen-activator-complexed forms, as probed by proteolytic susceptibility. *Eur J Biochem* **248**: 775–785

Foekens JA, Schmitt M, van Putten WL, Peters HA, Kramer MD, Janicke F and Klijn JG (1993) Plasminogen activator inhibitor-1 and prognosis in primary breast cancer. *J Clin Oncol* **12**: 1648–1658

Gils A, Knockaert I and Declerck PJ (1996) Substrate behavior of plasminogen activator inhibitor-1 is not associated with a lack of insertion of the reactive site loop. *Biochem* **35**: 7474–7481

Grondahl-Hansen J, Christensen IJ, Rosenquist C, Brunner N, Mouridsen HT, Dano K and Blichert-Toft M (1993) High levels of urokinase-type plasminogen activator and its inhibitor PAI-1 in cytosolic extracts of breast carcinomas are associated with poor prognosis. *Cancer Res* **53**: 2513–2521

Hofmann R, Lehmer A, Buresch M, Hartung R and Ulm K (1996) Clinical relevance of urokinase plasminogen activator, its receptor, and its inhibitor in patients with renal cell carcinoma. *Cancer* **78**: 487–492

Janicke F, Schmitt M, Pache L, Ulm K, Harbeck N, Hofler H and Graeff H (1993) Urokinase (uPA) and its inhibitor PAI-1 are strong and independent prognostic factors in node-negative breast cancer. *Breast Cancer Res Treat* **24**: 195–208

Kanse SM, Kost C, Wilhelm OG, Andreasen PA and Preissner KT (1996) The urokinase receptor is a major vitronectin binding protein on endothelial cells. *Exp Cell Res* **224**: 344–353

Kjoller L, Kanse SM, Kirkegaard T, Rodenburg KW, Ronne E, Goodman SL, Preissner KT, Ossowski L and Andreasen PA (1997) Plasminogen activator inhibitor-1 represses integrin- and vitronectin-mediated cell migration independently of its function as an inhibitor of plasminogen activation. *Exp Cell Res* **232**: 420–429

Kobayashi H, Fujishiro S and Terao T (1994) Impact of urokinase-type plasminogen activator and its inhibitor type 1 on prognosis in cervical cancer of the uterus. *Cancer Res* **54**: 6539–6548

Kuhn W, Pache L, Schmalfeldt B, Dettmar P, Schmitt M, Janicke F and Graeff H (1994) Urokinase (uPA) and PAI-1 predict survival in advanced ovarian cancer patients (FIGO III) after radical surgery and platinum-based chemotherapy. *Gynecol Oncol* **55**: 401–409

Kwaan HC (1992) The plasminogen-plasmin system in malignancy. *Cancer Metast Rev* **11**: 291–311

Levenson AS, Kwaan HC, Svoboda KM, Weiss IM, Sakurai S and Jordan VC (1998) Oestradiol regulation of the components of the plasminogen-plasmin system in MDA-MB-231 human breast cancer cells stably expressing the oestrogen receptor. *Br J Cancer* **78**: 88–95

Nekarda H, Siewert JR, Schmitt M and Ulm K (1994) Tumour-associated proteolytic factors uPA and PAI-1 and survival in totally resected gastric cancer. *Lancet* **343**: 117

Pedersen H, Grondahl-Hansen J, Francis D, Osterlind K, Hansen HH, Dano K and Brunner N (1994) Urokinase and plasminogen activator inhibitor type 1 in pulmonary adenocarcinoma. *Cancer Res* **54**: 120–123

Sancho E, Declerck PJ, Price NC, Kelly SM and Booth NA (1995) Conformational studies on plasminogen activator inhibitor (PAI-1) in active, latent, substrate, and cleaved forms. *Biochemistry* **34**: 1064–1069

Sier CFM, Vloedgraven HJM, Ganesh S, Griffion G, Quax PHA, Verheijen JH, Dooijewaard G, Welvaart K, van de Velde CJH, Lamers CBHW and Verspaget H (1994) Inactive urokinase and increased levels of inhibitor type 1 in colorectal cancer liver metastasis. *Gastroenterology* **10**: 1449–1456

Soff GA, Sanderowitz J, Gately S, Verrusio E, Weiss I, Brem S and Kwaan HC (1995) Expression of plasminogen activator inhibitor type 1 by human prostate carcinoma cells inhibits primary tumor growth, tumor-associated angiogenesis, and metastasis to lung and liver in an athymic mouse model. *J Clin Invest* **96**: 2593–2600

Stefansson S and Lawrence DA (1996) The serpin PAI-1 inhibits cell migration by blocking integrin alpha V beta 3 binding to vitronectin. *Nature* **383**: 441–443

Sugiura Y, Ma L, Sun B, Shimada H, Laug WE, Seeger RC and DeClerck Y (1999) The plasminogen-plasminogen activator (PA) system in neuroblastoma: role of PA inhibitor-1 in metastasis. *Cancer Res* **59**: 1327–1336

Verhamme I, Kvassman J, Day D, Debrock S, Vleufels N, Declerck PJ and Shore JD (1999) Accelerated conversion of human plasminogen activator inhibitor-1 to its latent form by antibody binding. *J Biol Chem* **274**: 17511–17517

Vleugels N, Gils A, Mannaerts S, Knockaert I and Declerck PJ (1998) Evaluation of the mechanism of inactivation of plasminogen activator-1 by monoclonal antibodies using a stable variant. *Fibrinolysis Proteolysis* **12**: 277–282

Waltz DA, Natkin LR, Fujita RM, Wei Y and Chapman HA (1997) Plasmin and plasminogen activator inhibitor type 1 promote cellular motility by regulating the interaction between the urokinase receptor and vitronectin. *J Clin Invest* **100**: 58–67

Wei Y, Waltz DA, Rao N, Drummond RJ, Rosenberg S and Chapman HA (1994) Identification of the urokinase receptor as an adhesion receptor for vitronectin. *J Biol Chem* **269**: 32380–32388

*© 2000 Cancer Research Campaign*
